# Geospatial Correlation of Amyopathic Dermatomyositis With Fixed Sources of Airborne Pollution: A Retrospective Cohort Study

**DOI:** 10.3389/fmed.2019.00085

**Published:** 2019-04-24

**Authors:** David R. Pearson, Victoria P. Werth

**Affiliations:** ^1^Department of Dermatology, University of Minnesota Medical School, Minneapolis, MN, United States; ^2^Corporal Michael J. Crescenz VA Medical Center, Philadelphia, PA, United States; ^3^Department of Dermatology, Perelman School of Medicine, University of Pennsylvania, Philadelphia, PA, United States

**Keywords:** dermatomyositis, pollution, environmental, geospatial analysis, Moran index

## Abstract

**Objective:** Dermatomyositis (DM) may result from exogenous triggers, including airborne pollutants, in genetically susceptible individuals. The United States Environmental Protection Agency's 2011 National Air Toxics Assessment (NATA) models health risks associated with airborne emissions, available by ZIP code tabulation area (ZCTA). Important contributors include point (fixed), on-road, and secondary sources. The objective of this study was to investigate the geospatial distributions of DM and subtypes, classic DM (CDM) and clinically amyopathic DM (CADM), and their associations with airborne pollutants.

**Methods:** This retrospective cohort study identified 642 adult DM patients from 336 unique ZCTAs. GeoDa v.1.10 was used to calculate global and local Moran's indices and generate local indicator of spatial autocorrelation (LISA) maps. All Moran's indices and LISA maps were permuted 999 times.

**Results:** Univariate global Moran's indices for DM, CDM, and CADM prevalence were not significant, but LISA maps demonstrated differential local spatial clustering and outliers. CADM prevalence correlated with point sources (bivariate global Moran's index 0.071, pseudo-*p* = 0.018), in contrast to CDM (−0.0053, pseudo-*p* = 0.46). Bivariate global Moran's indices for DM, CDM, and CADM prevalence did not correlate with other airborne toxics, but bivariate LISA maps revealed local spatial clustering and outliers.

**Conclusion:** Prevalence of CADM, but not CDM, is geospatially correlated with fixed sources of airborne emissions. This effect is small but significant and may support the hypothesis that triggering exposures influence disease phenotype. Important limitations are NATA data and ZCTA population estimates were collected from 2011 and ZCTA of residence may not have been where patients had greatest airborne pollutant exposure.

## Introduction

The pathogenesis of dermatomyositis (DM) is incompletely understood, but may result from diverse exogenous exposures that trigger disease in genetically susceptible individuals, including drugs, infections, and environmental factors such as ultraviolent radiation (UVR) and airborne pollutants (1–18). Important subtypes include classic DM (CDM), which demonstrates clinical and laboratory evidence of myositis, and clinically amyopathic DM (CADM), diagnosed when patients do not have symptomatic myositis and have only minimal or no objective findings of myositis (19, 20). There is evidence that disease phenotype has been associated with specific exposure patterns (5, 8, 10, 21).

The United States Environmental Protection Agency (EPA), an independent agency of the United States federal government that aims to protect human health and the environment, provides publicly available data on airborne emissions in the United States at the level of ZIP code tabulation areas (ZCTA) (22). ZCTAs are geographic representations of the United States Postal Service ZIP code service areas, and were developed by the United States Census Bureau to tabulate summary statistics over geographic areas. In 2015, the EPA released the most recent version of the National Air Toxics Assessment (NATA) based on 2011 emissions. NATA is a screening tool designed to provide information on potential risks of breathing air toxics (22). Emission levels of 181 air toxics (such as benzene, formaldehyde, and diesel particulate matter) were measured, then modeled to predict overall health risks and applied over geographic areas (22). Important contributors include *point* sources, which denote emissions from larger, stationary industrial and commercial facilities, *on-road* sources, produced by vehicles on roads, and *secondary* sources, which are formed from the chemical reactions of other emitted pollutants (22). Since DM has been associated with airborne pollutants, this dataset may be useful in analyzing exposure patterns (11–13).

Geospatial analysis is a statistical approach to analyze data over a geographic region, with applications in environmental science, public health, and other fields. Geospatial statistical techniques, including global and local Moran's indices, can be used to evaluate clustering and dispersion patterns, and have found increasing use in medicine (23–25). These techniques have not been applied to the study of DM due in part to the low incidence and prevalence of disease. The relatively large cohort at the University of Pennsylvania provides an opportunity to use geospatial analysis to assess exposure patterns in DM.

The objective of this study was to investigate the geospatial distributions of DM and its subtypes and their associations with airborne pollutants in the greater Philadelphia metropolitan area.

## Methods

### Patient Selection

This retrospective cohort study spanned January 1, 2000 through December 31, 2017. Data collection was complete July 31, 2018. Adult patients ≥18 years of age seen in dermatology or rheumatology clinics at the University of Pennsylvania with a United States ZCTA of primary residence listed in their medical record and a diagnosis of DM, encoded by International Classification of Disease (ICD) 9th and 10th revision codes (710.3 and M33.0X, M33.1X, M33.9X, respectively) were included. Patients were identified using PennSeek, a custom, secure implementation of Oracle's web-based Endeca Information Discovery platform adapted by the University of Pennsylvania Data Analytics Core. PennSeek allows for targeted keyword searches using Boolean logic of unstructured or semi-structured medical documents that reside in the main Penn Medicine electronic health record and ancillary systems, described in detail elsewhere (26). After assembling the cohort, individual patient charts were reviewed to verify diagnosis, exclude erroneously coded patients, and extract variables of interest.

### 2011 NATA Dataset

2011 NATA risk estimates including total airborne, point, on-road, and secondary source risks were taken from the EPA's dataset, organized by ZCTA (22).

### Variables

DM subtype was assigned based on the treating clinician's note. In cases of ambiguity or disagreement, evidence of myositis was determined by patient-reported symptoms plus an objective sign (elevated creatinine kinase or aldolase or consistent EMG, MRI, or muscle biopsy). Patients who fulfilled these criteria were labeled as CDM and those lacking labeled as CADM. Demographics and ZCTAs were extracted from the medical record. Prevalence was calculated using United States Census Bureau (USCB) 2007-2011 American Community Survey population estimates, organized by ZCTA (27).

### Analysis

Continuous variables are presented as median (interquartile range, IQR) and categorical data are presented as count (percentage). Heatmaps were created with Tableau Public version 10.4 (Tableau Software, Inc. 2018. Seattle, Washington).

Geospatial analysis was performed by calculation of the global Moran's index, an inferential statistic which estimates spatial autocorrelation and falls between −1 and +1 (28). More positive or negative values correspond to greater spatial clustering or competitive dispersion, respectively (28). To test the null hypothesis of random spatial distribution, empiric significance is computed by permutation to yield a pseudo-*p*-value (28). A pseudo-*p*-value differs from an analytic *p*-value because it is a summary of the results of the reference distribution, and is dependent on the number of permutations used (28). In order to visualize spatial clustering, local indicators of spatial autocorrelation (LISA) maps were created using local Moran's index calculations (29). This statistic identifies significant locations as high-high or low-low spatial clusters and high-low or low-high spatial outliers relative to neighboring regions (29). In both indices, neighbor less regions are excluded from analysis.

Bivariate global and local Moran's indices were calculated in the same fashion; global indices measure spatial autocorrelation between an outcome variable in one geographic region and a second variable in neighboring regions, and local indices were mapped with bivariate LISA (BiLISA) maps (30).

GeoDa version 1.10 was used for geospatial analyses. Shapefiles were obtained from the USCB 2017 ZCTA boundary files and the weights matrix was assigned first-order queen contiguity (neighboring regions share common edges or vertices). All global and local univariate and bivariate Moran's indices and LISA and BiLISA maps were permuted 999 times. A pseudo-*p* < 0.05 was set as significant.

### Ethics

This study was carried out in accordance with the recommendations of the University of Pennsylvania Institutional Review Board (protocol number 828959), which waived the requirement of written informed consent from subjects. Written consent was waived due to the retrospective nature of the study and minimal risk it posed to subjects. The protocol for this study conforms to the ethical guidelines of the Declaration of Helsinki. The protocol was approved by the University of Pennsylvania Institutional Review Board.

## Results

Of 205,084 adult patients seen in dermatology or rheumatology clinics at the University of Pennsylvania from January 1, 2000 through December 31, 2017, PennSeek identified 653 patients. Upon individual chart review, 9 patients were excluded for diagnoses other than DM and 2 were excluded for absence of a ZCTA in their medical record, resulting in 642 patients who met inclusion criteria.

Baseline characteristics of the patient cohort are noted in Table 1. The median age at symptom onset was 49 (IQR 37, 58) years. Most patients were female (532, 82.8%), white (476, 74.1%), and diagnosed with CDM (439, 68.4%), though a notable fraction had CADM (203, 31.6%). Data on year of symptom onset was available for 630 patients (98.1%) and divided into approximate quartiles. One hundred fifty-nine patients (25.2%) developed symptoms in 2005 or earlier, 176 patients (27.9%) between 2006 and 2009, 153 patients between 2010 and 2013, and 142 patients (22.5%) from 2014 to the end of the study period.

**Table 1:** Baseline characteristics of adult dermatology and rheumatology patients with dermatomyositis (DM) seen at the University of Pennsylvania between January 1, 2000 and December 31, 2017, with median 2011 NATA risk calculations.

**Metric**	**Cohort (*n* = 642)**
Median age at symptom onset, years (IQR)	49 (37, 58)
Female sex	532 (82.8%)
**PATIENT-REPORTED RACE/ETHNICITY**	
White	476 (74.1%)
Black	76 (11.8%)
Asian, American Indian, or Alaskan Native	20 (3.1%)
Other	70 (10.9%)
**DM SUBTYPE**	
CDM	439 (68.4%)
CADM	203 (31.6%)
**SYMPTOM ONSET YEAR QUARTILES (*****n*** **=** **630)**	
2005 or earlier	159 (25.2%)
2006–2009	176 (27.9%)
2010–2013	153 (24.3%)
2014–2017	142 (22.5%)
**ZCTA**	
Unique ZCTAs	336
2010 Census population	8,110,198
Median DM prevalence per ZCTA (IQR), per 100,000	8.6 (4.6, 15.2)
Median DM prevalence per Phl metropolitan ZCTA (IQR), per 100,000 (*n* = 607)	9.2 (5.2, 16.3)
**MEDIAN 2011 NATA RISK PER ZCTA, PER MILLION**	
Total airborne risk (IQR)	43 (37, 48)
Point source risk (IQR)	1.24 (0.64, 1.67)
On-road source risk (IQR)	9.13 (6.56, 12.18)
Secondary source risk (IQR)	18.10 (16.47, 19.17)

*CADM, clinically amyopathic dermatomyositis; CDM, classic dermatomyositis; DM, dermatomyositis; IQR, interquartile range; NATA, National Air Toxics Assessment; Phl, Philadelphia; ZCTA, zip code tabulation area*.

Three hundred thirty-six unique ZCTAs represented a total underlying population of 8,110,198 (Table 1, Data Sheet 1). The median prevalence of DM per ZCTA was 8.6 (IQR 4.6, 15.2) per 100,000. When ZCTAs outside of the greater Philadelphia metropolitan area were excluded, the median prevalence of DM was 9.2 (IQR 5.2, 16.3) per 100,000. The median total airborne risk per ZCTA was 43 (IQR 37, 48) per million, including 1.24 (IQR 0.64, 1.67) per million from point sources, 9.13 (IQR 6.56, 12.18) per million from on-road sources, and 18.10 (IQR 16.47, 19.17) per million from secondary sources.

**Figure 1:**
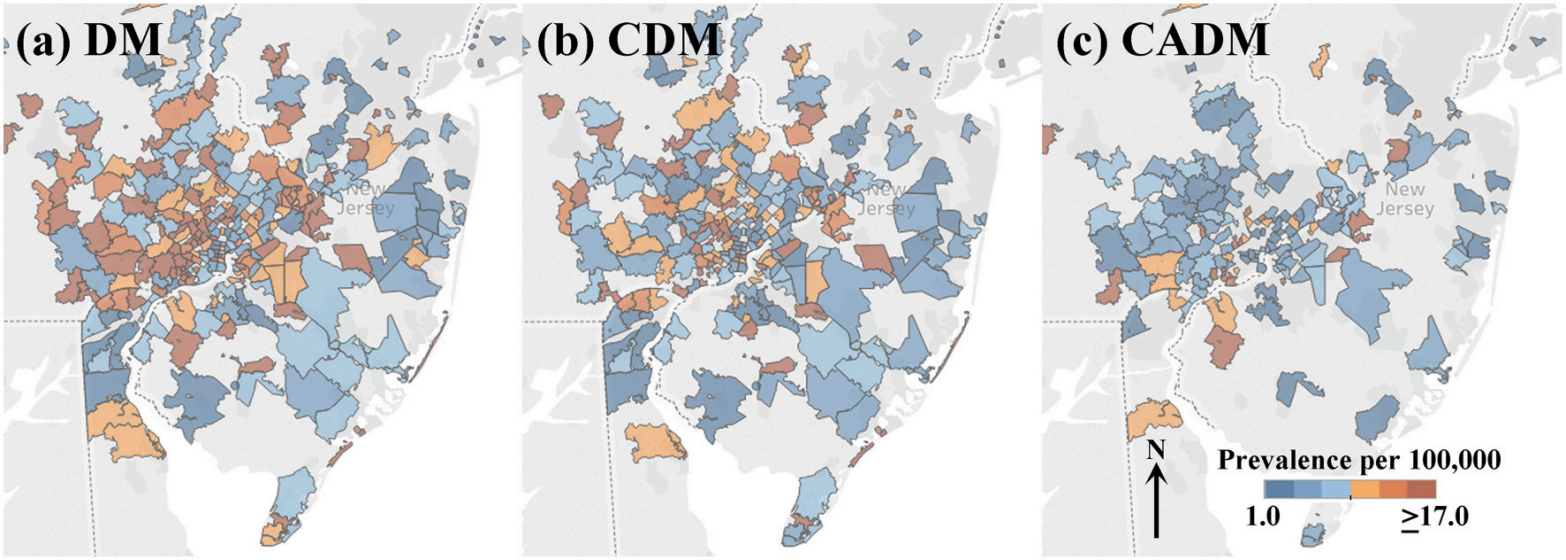
Heatmaps of prevalence per 100,000 of the full cohort of dermatomyositis (DM) patients **(a)**, and subtypes classic DM (CDM, **b**) and clinically amyopathic DM (CADM, **c**) in the greater Philadelphia metropolitan area (legend inset: low prevalence, blue; high prevalence, orange). Many ZCTAs with high prevalence of DM and CDM are observed in the western and northern metropolitan area. CADM prevalence is lower across the region and aligns along a northeast-southwest axis.

Heatmaps of the prevalence of DM and subtypes CDM and CADM in the greater Philadelphia metropolitan area are illustrated in Figure 1, divided into sextiles. Many ZCTAs with high prevalence of DM and CDM were observed in the western and northern metropolitan area. CADM prevalence is lower overall and aligns along a northeast-southwest axis. Figure S1 shows heatmaps of the onset year by quartile, with no clear patterns of clustering or outliers.

### Geospatial Analysis

**Figure 2:**
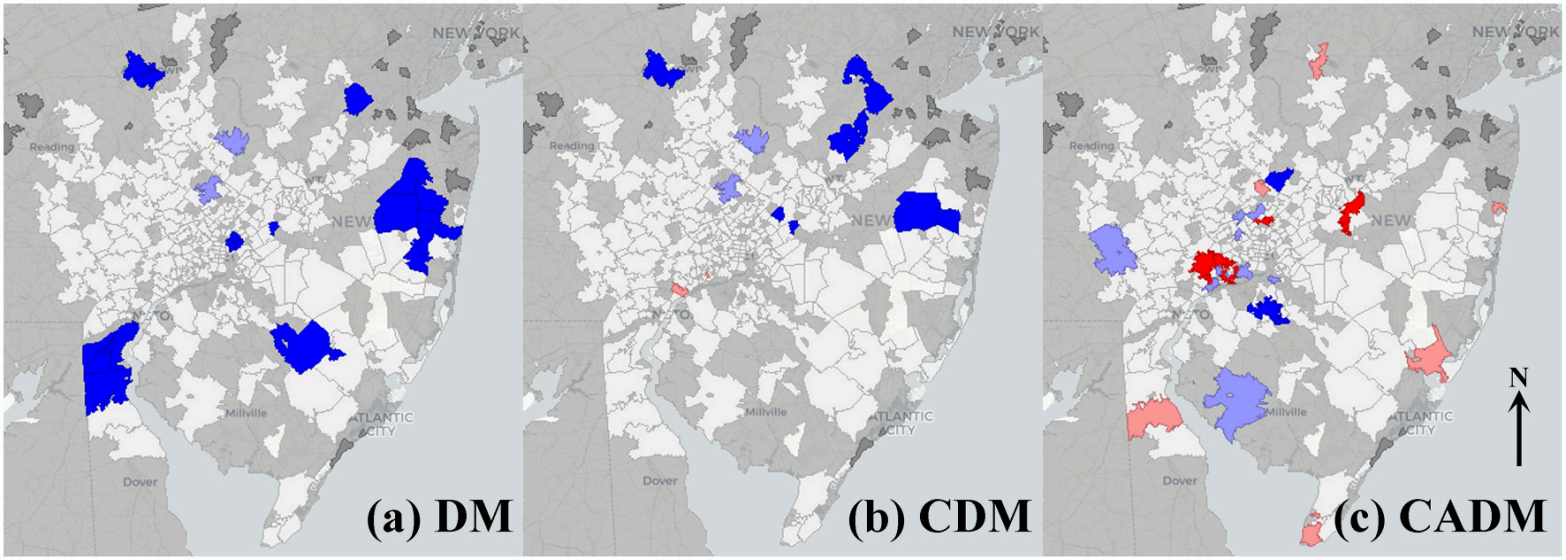
Local indicators of spatial autocorrelation (LISA) maps for prevalence of the full cohort of dermatomyositis (DM) patients **(a)**, and subtypes classic DM (CDM, **b**) and clinically amyopathic DM (CADM, **c**). High-high (red) and low-low (bright blue) spatial clusters, and high-low (pink) and low-high (light blue) spatial outliers relative to neighboring regions are identified. Geospatially non-significant (white) and neighborless (dark gray) regions are visible. Differential spatial clustering is most notable between CDM **(b)** and CADM **(c)**.

Univariate global Moran's indices for the prevalence of DM, CDM, and CADM were not significant (Table 2), but LISA maps demonstrated differential local spatial clustering and outliers (Figure 2), as indicated by the presence of high-high (red) and low-low clustering (bright blue), and high-low (pink) and low-high (light blue) outliers. DM and CDM shared several clustered and outlier ZCTAs, and differed notably from CADM, which demonstrated high-high clustering along a northeast-southwest axis. High-low outliers occurred predominantly in ZCTAs of low relative population.

**Table 2:** Univariate global Moran's indices of the prevalence of dermatomyositis (DM) and subtypes, and bivariate global Moran's indices of DM prevalence vs. 2011 NATA metrics.

**Outcome variable**	**Lagged variable**	**Global Moran's index**	**Pseudo *p*-value**
DM prevalence	Univariate	0.0054	0.16
	Total airborne risk^*^	0.0080	0.34
	Point sources^*^	0.0019	0.36
	On-road sources^*^	0.0057	0.15
	Secondary sources^*^	0.0036	0.46
CDM prevalence	Univariate	0.0051	0.16
	Total airborne risk^*^	0.0080	0.33
	Point sources^*^	−0.0053	0.46
	On-road sources^*^	0.0082	0.28
	Secondary sources^*^	−0.00076	0.47
CADM prevalence	Univariate	0.064	0.06
	Total airborne risk^*^	−0.0015	0.49
	Point sources^*^	**0.071**	**0.02**
	On-road sources^*^	−0.025	0.18
	Secondary sources^*^	0.043	0.09

*CADM, clinically amyopathic dermatomyositis; CDM, classic dermatomyositis; DM, dermatomyositis; NATA, National Air Toxics Assessment*.

* *Bivariate global Moran's index*.

*Bold values indicate significant finding*.

**Figure 3:**
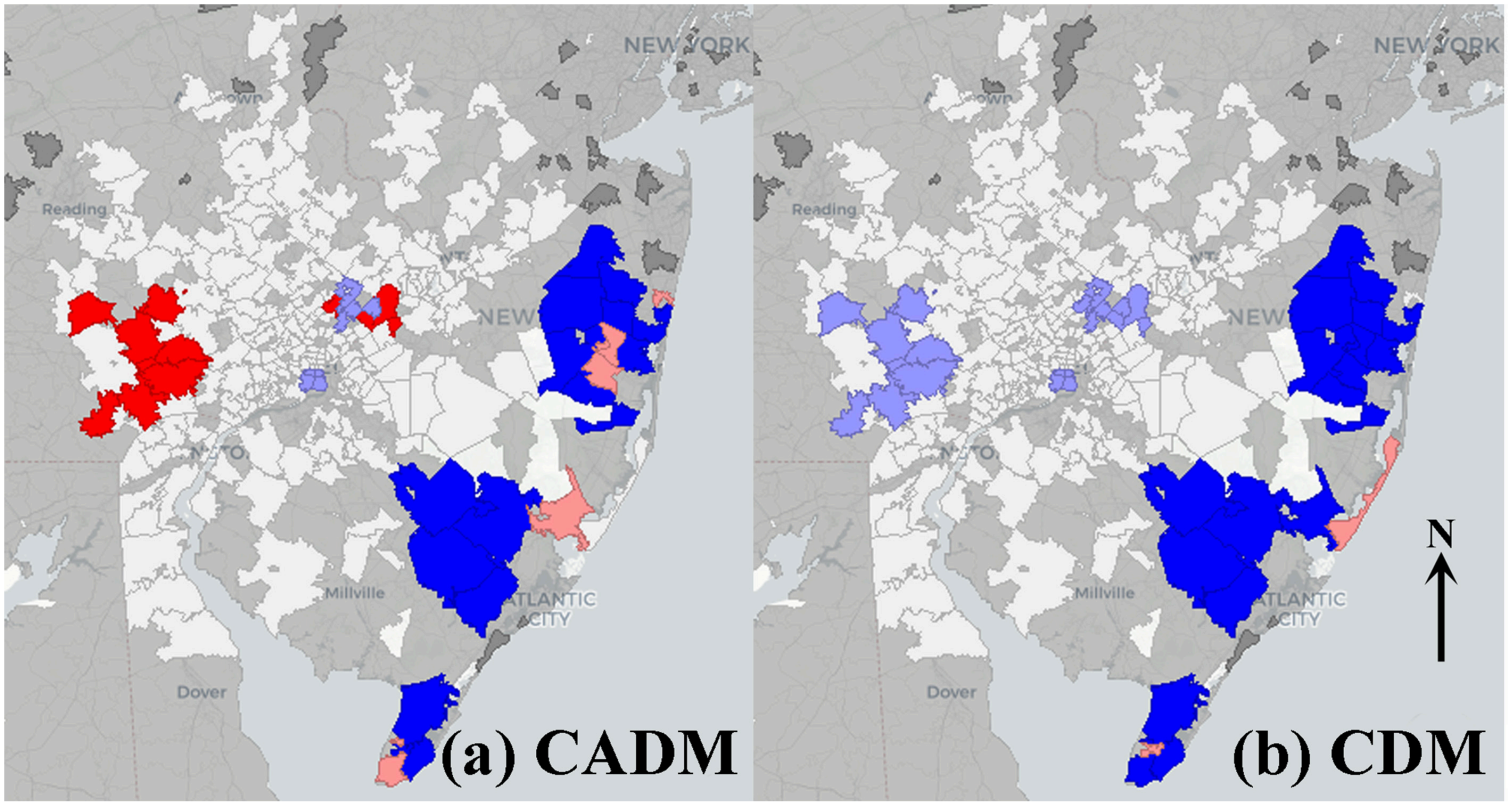
Bivariate local indicators of spatial autocorrelation (BiLISA) maps for prevalence of clinically amyopathic dermatomyositis (CADM, **a**) and classic dermatomyositis (CDM, **b**) vs. point sources. High-high (red) and low-low (bright blue) spatial clusters, and high-low (pink) and low-high (light blue) spatial outliers relative to neighboring regions are identified. In CADM **(a)**, western metropolitan high-high clustering and eastern metropolitan low-low clustering is prominent, while in CDM **(b)**, western low-high outliers and eastern low-low clustering is notable. Geospatially non-significant (white) and neighbor less (dark gray) regions are visible.

The prevalence of CADM vs. point sources showed a significant bivariate global Moran's index (0.071, pseudo-*p* = 0.02), and BiLISA mapping demonstrating western metropolitan high-high clustering and eastern metropolitan low-low clustering (Figure 3a). In contrast, the prevalence of CDM vs. point sources was not significant (−0.0053, pseudo-*p* = 0.46), and BiLISA mapping demonstrating western low-high outliers and eastern low-low clustering (Figure 3b). Bivariate global Moran's indices for the prevalence of DM, CDM, and CADM vs. other airborne toxics were not significant, and BiLISA mapping revealed similar local spatial clustering and outliers between subtypes (Figure S2).

Univariate global Moran's indices for both DM onset year (by quartile) and median age at symptom onset were not significant (data not shown).

## Discussion

In this retrospective study of 642 DM patients, we found a significant geospatial correlation between exposure to point sources of airborne pollutants and CADM. Other airborne toxics including overall calculated airborne risk, on-road, and secondary sources did not correlate with DM, CDM, or CADM prevalence.

The association between point sources and CADM is modest but significant, as seen by the magnitude of the global Moran's index. Since DM has been associated with a variety of exogenous triggers besides airborne toxics, it is not surprising that this exposure does not account for all cases. It is noteworthy that CDM was not correlated with point sources, supporting the hypothesis that the type of triggering exposure may influence disease phenotype.

Past studies have demonstrated associations between specific exogenous triggers and serotype, which is known to correlate with clinical phenotype (31–33). The intensity of UVR exposure has been strongly associated with the proportion of CDM patients expressing anti-Mi-2 antibodies (odds ratio [OR] 6.0), while anti-TIF1-γ antibodies have been negatively correlated with latitude (OR 0.96) (8, 21). HLA alleles associated with anti-Mi-2 and anti-TIF1-γ antibodies were also negatively associated with latitude (21). Anti-MDA-5 antibodies were inversely associated with population of city of residence and appeared to cluster along rural areas near the Kiso River in Japan (10). Hydroxyurea may induce a DM-like eruption that spares muscles, while other drugs may induce a CDM-like presentation and viral infections may trigger juvenile DM (5, 34).

This study has important limitations. It is a retrospective cohort from a tertiary referral facility in a heavily-populated urban center. The ZCTA of residence listed in the patient's chart may not have been the location where greatest airborne pollutant exposure occurred and does not factor transportation routes or job location. The listed ZCTA may not have been where DM onset occurred, especially in longstanding cases. Reference data were taken from available years: ZCTAs were collected from medical records in 2018, ZCTA shapefiles were based on 2017 USCB boundary files, NATA metrics were calculated from data collected in 2011, and ZCTA population estimates were collected from 2007 to 2011 in the USCB American Community Survey. Both population estimates and airborne pollutant levels may have changed since 2011, though more recent, reliable data was not available.

It is important to note that NATA metrics are risk assessments based on collected airborne emissions and do not reflect exposure to all compounds, all pathways of exposure, or accurately model episodic emissions (22). Risk assessments by source represent pooled data from many pollutants, and the contributed risks of individual compounds were not assessed. Furthermore, these data apply to geographic areas and groups, rather than specific locations or individuals (22).

The prevalence of DM found in this study is lower than previously reported (35). This estimate is not population-based and likely underestimates the true prevalence in the greater Philadelphia metropolitan area. During the 18-year time period of this study, there were 5 major academic medical centers and a large number of group and individual dermatology and rheumatology practices, many of whom see DM patients not included here.

While the spatial distribution of onset of DM symptoms changed over time, specific regions did not cluster by onset year. DM frequency remained approximately stable when comparing the 4-year intervals from 2006 to 2009, 2010 to 2013, and 2014 to 2017. However, DM, especially CADM, is frequently delayed in diagnosis or misdiagnosed and presentation to a tertiary referral center may result in additional delay, so patients with more recent onset may be unaccounted for (36).

Identifying exogenous triggers for DM is challenging given the heterogeneity of both exposure and the underlying disease. Geospatial analysis is a candidate method for studying geographic patterns of exposure and may have applications examining infectious triggers, pollutants, demographics, and other factors. Future population-based studies may better estimate these associations. Further exploration of the geospatial distribution of DM phenotype by serology, malignancy association, or the presence of interstitial lung disease may also be useful for determining risk factors and understanding disease pathogenesis.

In conclusion, in this retrospective cohort study we found prevalence of CADM, but not CDM, is geospatially correlated with larger, geographically fixed sources of airborne pollution. This effect is small but significant and may support the hypothesis that triggering exposures may influence disease phenotype in DM.

## Ethics Statement

This study was carried out in accordance with the recommendations of the University of Pennsylvania Institutional Review Board (protocol number 828959), which waived the requirement of written informed consent from subjects. Written consent was waived due to the retrospective nature of the study and minimal risk it posed to subjects. The protocol for this study conforms to the ethical guidelines of the Declaration of Helsinki. The protocol was approved by the University of Pennsylvania Institutional Review Board.

## Author Contributions

DP and VW contributed conception and design of the study. DP collected the primary data, performed the statistical analysis, and wrote the first draft of the manuscript. DP and VW contributed to manuscript revision and read and approved the submitted version.

### Conflict of Interest Statement

The authors declare that the research was conducted in the absence of any commercial or financial relationships that could be construed as a potential conflict of interest.

## Supplementary Material

Data Sheet 1 Unique ZCTAs and corresponding census populations and DM counts, calculated prevalences, and NATA risk estimates are shown. On the far right, counts of DM are listed by year of symptom onset. CADM, clinically amyopathic dermatomyositis; CDM, classic dermatomyositis; DM, dermatomyositis; NATA, National Air Toxics Assessment; ZCTA, zip code tabulation area.

Figure S1 Heatmaps of new-onset cases of dermatomyositis in the greater Philadelphia metropolitan area in 2005 or earlier **(a)**, 2006–2009 **(b)**, 2010-2013 **(c)**, and 2014–2017 **(d)** (legend inset: lower count, light blue; higher count, dark blue). Geospatial analysis did not demonstrate clustering or dispersion.

Figure S2 Bivariate local indicators of spatial autocorrelation (BiLISA) maps for prevalence of the full cohort of dermatomyositis (DM) and subtypes classic DM (CDM) and clinically amyopathic DM (CADM) vs. total airborne risk (**a–c**, respectively), on-road sources (**d–f**, respectively), and secondary sources (**g–I**, respectively). High-high (red) and low-low (bright blue) spatial clusters, and high-low (pink) and low-high (light blue) spatial outliers relative to neighboring regions are identified. Geospatially non-significant (white) and neighbor less (dark gray) regions are visible. Note the similarities in both spatial clustering and outliers between DM, CDM, and CADM. All global Moran's indices were non-significant.
