# Supplementary figures and images for: Geospatial Correlation of Amyopathic Dermatomyositis With Fixed Sources of Airborne Pollution: A Retrospective Cohort Study
Source: Front Med (Lausanne). 2019 Apr 24;6:85. doi: 10.3389/fmed.2019.00085 (PMC6491706; doi:10.3389/fmed.2019.00085)

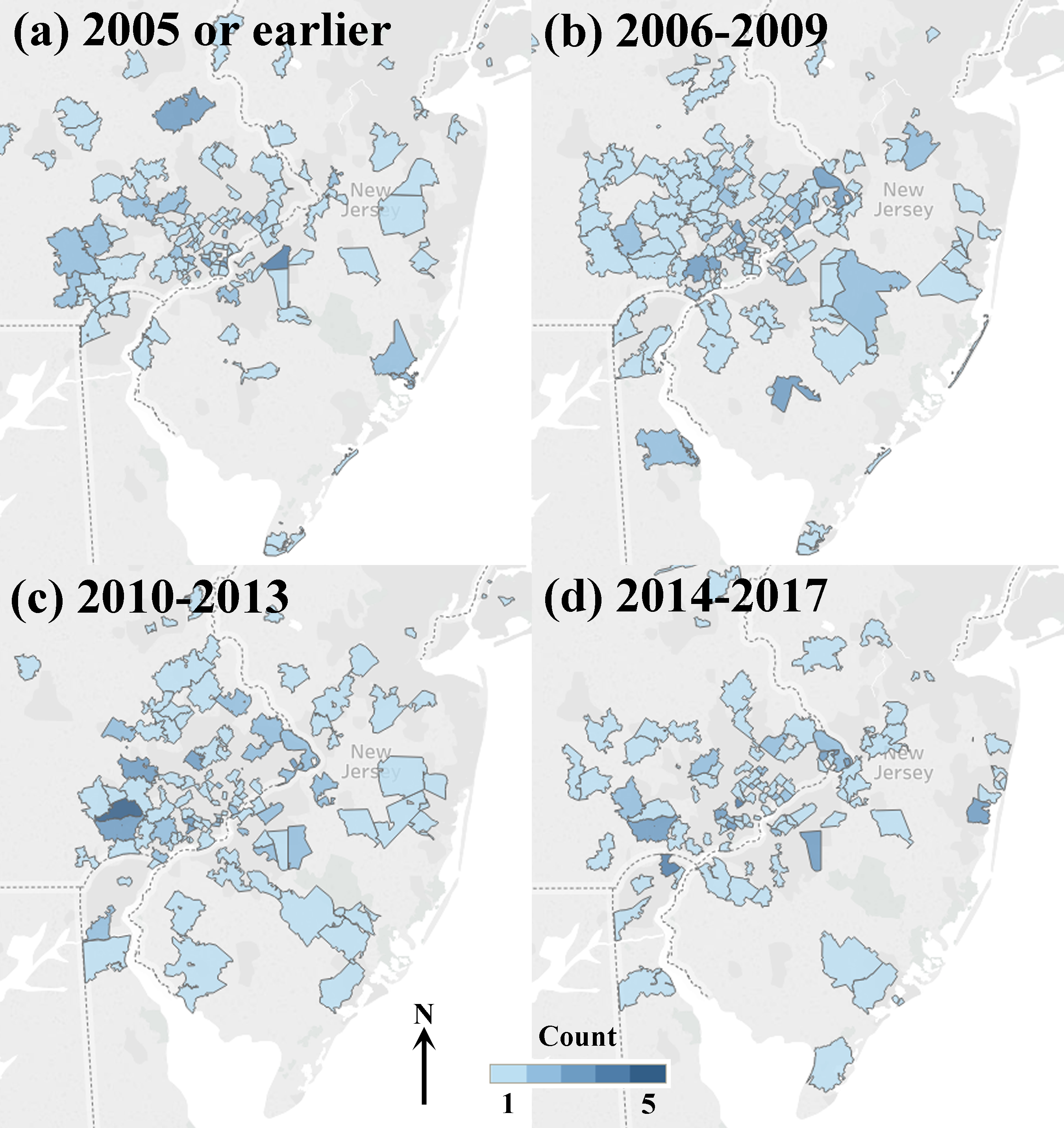

Supplement: Figure S1 — Heatmaps of new-onset cases of dermatomyositis in the greater Philadelphia metropolitan area in 2005 or earlier (a), 2006–2009 (b), 2010-2013 (c), and 2014–2017 (d) (legend inset: lower count, light blue; higher count, dark blue). Geospatial analysis did not demonstrate clustering or dispersion. [file Image_1.PNG]

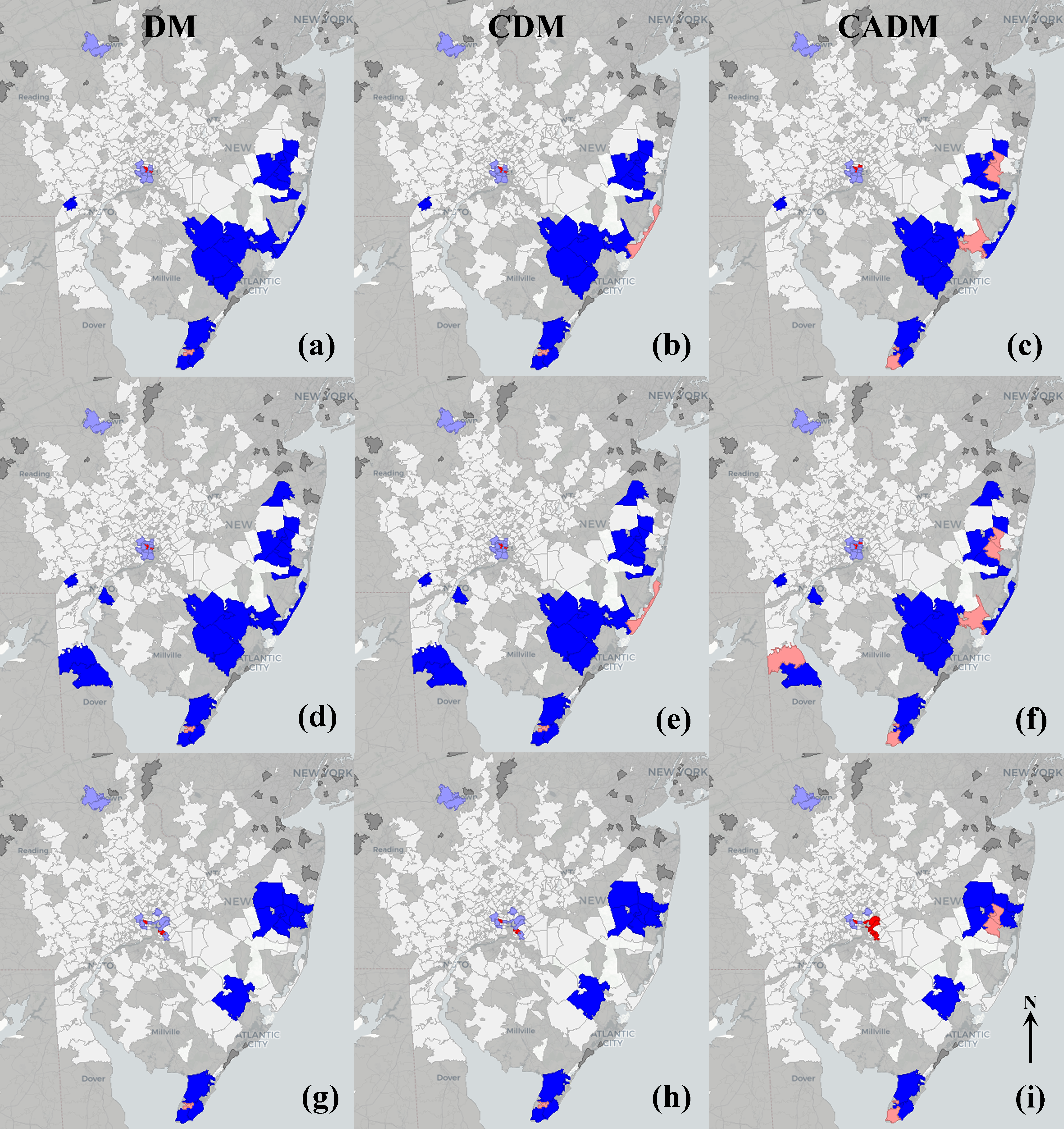

Supplement: Figure S2 — Bivariate local indicators of spatial autocorrelation (BiLISA) maps for prevalence of the full cohort of dermatomyositis (DM) and subtypes classic DM (CDM) and clinically amyopathic DM (CADM) vs. total airborne risk (a–c, respectively), on-road sources (d–f, respectively), and secondary sources (g–I, respectively). High-high (red) and low-low (bright blue) spatial clusters, and high-low (pink) and low-high (light blue) spatial outliers relative to neighboring regions are identified. Geospatially non-significant (white) and neighbor less (dark gray) regions are visible. Note the similarities in both spatial clustering and outliers between DM, CDM, and CADM. All global Moran's indices were non-significant. [file Image_2.PNG]
